# The role of validation in establishing the scientific credibility of predictive toxicology approaches intended for regulatory application

**DOI:** 10.1016/j.comtox.2020.100144

**Published:** 2021-02

**Authors:** Eann A. Patterson, Maurice P. Whelan, Andrew P. Worth

**Affiliations:** a School of Engineering, University of Liverpool, Liverpool, UK; b European Commission, Joint Research Centre (JRC), Ispra, Italy

**Keywords:** Predictive toxicology approach, Credibility, Validation, Toxicological relevance, Toxicological reliability

## Abstract

New approaches in toxicology based on in vitro methods and computational modelling offer considerable potential to improve the efficiency and effectiveness of chemical hazard and risk assessment in a variety of regulatory contexts. However, this presents challenges both for developers and regulatory assessors because often these two communities do not share the same level of confidence in a new approach. To address this challenge, various assessment frameworks have been developed over the past 20 years with the aim of creating harmonised and systematic approaches for evaluating new methods. These frameworks typically focus on specific methodologies and technologies, which has proven useful for establishing the validity and credibility of individual methods. However, given the increasing need to compare methods and combine their use in integrated assessment strategies, the multiplicity of frameworks is arguably becoming a barrier to their acceptance. In this commentary, we explore the concepts of model validity and credibility, and we illustrate how a set of seven credibility factors provides a method-agnostic means of comparing different kinds of predictive toxicology approaches. It is hoped that this will facilitate communication and cross-disciplinarity among method developers and users, with the ultimate aim of increasing the acceptance and use of predictive approaches in toxicology.

## Highlights

• The role of validation in establishing the credibility of predictive methods is discussed.
• Various assessment frameworks for predictive methods have evolved independently, being developed by different communities.
• A set of seven credibility factors is proposed as a method-agnostic means of comparing the various assessment frameworks.
• It is hoped this will facilitate communication and cross-disciplinary collaboration between method developers and users.

## 1 Introduction

Non-animal approaches to toxicology based on in vitro and computational methods are gaining importance in the regulatory domain. Reasons for this include the possibility to reduce animal testing, deliver more target-species specificity and sensitivity, and generate data in high throughput and high content formats to save time and reduce costs. Such methods can also provide the type of mechanistic information being increasingly used in chemical hazard assessment, for example to identify substances of concern such as developmental neurotoxicants [1] and endocrine disruptors [2]. Nevertheless, the acceptance and uptake of new ‘predictive’ toxicology methods has been slow, primarily because the method development and regulatory assessment communities typically do not share the same level of confidence in a new approach.

To address this issue, a number of developments started in the 1990s to establish internationally recognised frameworks for the validation of new test methods with the expectation that this would overcome the barriers to their regulatory acceptance [3]. These efforts led to internationally agreed validation principles and formed the basis of current (2005) international guidance on the validation of new test methods [4], as well as the modular validation approach [5] applied by the JRC's European Union Reference Laboratory for Alternatives to Animal Testing (EURL ECVAM). The emphasis at the time was on in vitro methods, since these were considered to be the most mature of the new methods. Subsequently, validation principles originally conceived for experimental test methods were re-interpreted and adapted for computational methods based on quantitative structure-activity relationships (QSARs), and technical guidance was developed on how to apply these QSAR validation principles [6]. In parallel, assessment frameworks for physiologically based kinetic (PBK) models were also being developed [7].

Recent years have seen a progressive blurring of the traditional distinction between experimental and computational approaches in toxicology. Experimental methods have become increasingly sophisticated and often rely on complex computational processing and analysis of the large amounts of data they generate. Likewise, computational methods based on mathematical modelling and machine learning rely heavily on the availability of large experimental datasets for parameterisation and validation. Individual methods are also being combined and applied within Integrated Approaches to Testing and Assessment (IATA) [8] in order to expand chemical applicability domains and to address more complex toxicological endpoints. Given the trend towards method integration, the regulatory community has developed an increasing number of guidance documents on relevant topics such as data quality, data integration, uncertainty characterisation, and weight of evidence [9].

As a consequence of these developments, the regulatory community is now confronted not only with an increasingly diverse and sophisticated range of new methods, but also a growing number of assessment frameworks.

In this commentary, we argue that the various method-centric frameworks have a lot of features in common, and we propose a method-agnostic set of credibility factors that could be used to compare different types of predictive toxicology approaches. To set the scene for the proposed set of credibility factors, we first outline the key features of validation frameworks already developed for QSAR models, defined approaches for skin sensitisation, in vitro DNT methods, and PBK models. We then illustrate the relationships between the credibility factors and the four validation frameworks. It is hoped that the proposed credibility factors will facilitate communication and cross-disciplinary collaboration among method developers and users, with the ultimate aim of increasing the acceptance and use of predictive approaches in toxicology.

## 2 Validation principles and frameworks

In regulatory toxicology, validation has been defined as “the process by which the reliability and relevance of a particular approach, method, process or assessment is established for a defined purpose” [4]. It is predominantly a scientific pursuit that seeks to objectively and independently characterise the performance of an approach within prescribed operating conditions that reflect its anticipated uses [10]. Since the OECD definition of validation is rather general, its translation into practical validation frameworks can vary, depending on circumstance. For example, at one end of the “applications spectrum”, validated test methods that are intended for inclusion in OECD Test Guidelines need to be applicable to a wide range of chemicals, and make predictions for standard toxicological endpoints. At the other end, methods that are used to support the efficacy and safety of drugs or biological therapies can be highly specialised, addressing bespoke applications. In both cases, validation is carried out for a “defined purpose” but the nature and scope of the purpose varies.

Historically, different validation frameworks have been developed somewhat independently by different communities [11], as illustrated by the different sets of principles or criteria shown in Table 1. A notable feature of these different frameworks is that they relate primarily to one particular type of method (i.e. in vivo tests, in vitro assays, QSARs). This can be seen as an illustration of the disciplinary silos that result from compartmentalisation of methods across the sciences [12].

**Table 1:** Principles/criteria of different validation frameworks employed within the toxicology community.

Minimum criteria for a valid test	ECVAM principles on test validity	QSAR validation principles	Defined Approaches	In vitro Developmental Neurotoxicity methods	Physiologically based kinetic models
OECD, 2005 [4]	Hartung et al, 2004 [5]	OECD, 2007 [6]	OECD 2016, 2017 [8], [14], [20]	Bal-Price et al, 2018 [22]	OECD Guidance Document [in progress]
**Rationale available** for scientific need and regulatory purpose **Relevance**: relationship of test endpoint to in vivo biological effect **Protocol available**: subjected to independent peer-review **Repeatability and reproducibility shown**: intra-test, intra and inter-lab variability defined **Reference performance demonstrated** using reference chemicals **Toxicity performance** evaluated against existing relevant toxicity data **Validation available**: all data supporting assessment of validity available for review **Good Laboratory Practice** used to obtain data	**Test method definition**: endpoint, training set, prediction model (PM), applicability and mechanism **Within-laboratory variability:** assessment of reproducibility of data **Transferability**: confirmation by second operator (facility) **Between-laboratory variability**: assessment of reproducibility in 2 to 4 laboratories **Predictive capacity**: ability to predict beyond training set based on comparisons **Applicability domain**: definition of chemical classes and/or ranges for which predictions are reliable **Performance standards**: reference chemicals defined for equivalence between original and new (similar) tests	**A defined endpoint:** transparency of effect being predicted **An unambiguous algorithm:** transparency of description of an unambiguous model **A defined applicability domain:** recognising QSARs are reductionist and inevitably limited to subsets of chemical space **Appropriate measures of goodness-of-fit, robustness & predictivity:** performance when using training set or test set **A mechanistic interpretation:** an assessment of mechanistic associations between descriptors and end-points	**Structure:** elements of defined approach, information provided: **Relevance:** mechanistic basis **Predictive Capacity:** performance compared to reference data **Reliability:** reproducibility **Applicability domain:** technical limitations and chemical space **Complexity** of the Data Interpretation Procedure **Transparency:** availability of elements	**Test system:** definition, stability and biological relevance of cell-based system **Exposure scheme:** details of chemical treatment and incubation conditions **Documentation / SOP:** transparency in method protocol **Endpoint(s):** transparency of effect(s) being measured **Test method controls:** chemicals used to determine whether effects are positive or negative, and endpoint-specific **Data evaluation:** statistical analysis of concentration–response data **Testing strategy:** role in test battery **Robustness:** reproducibility within and between labs and over time **Test benchmarks:** sensitivity and specificity, data acceptance criteria **Prediction model:** how to extrapolate the in vitro data **Applicability domain:** chemistry and biological pathways **Screening hits:** definition of positive vs negative response	**Biological basis:** physiologically relevant model structure and parameters **Theoretical basis of model equations:** established mathematical basis such as Michaelis-Menten kinetics **Reliability of input parameters:** reproducibility **Sensitivity of output to input parameters:** relative importance of input parameters in determining simulation outcome **Goodness-of-fit and predictivity:** performance when using training set or test set

### 2.1 Integrated approaches to testing and assessment (IATA)

The need to use data from multiple methods to carry out a safety assessment has been recognised for over 20 years [13], and has led to the concept of Integrated Approaches to Testing and Assessment (IATA). According to OECD guidance [14], an IATA is an “approach based on multiple information sources used for the hazard identification, hazard characterisation and/or safety assessment of chemicals. An IATA integrates and weights all relevant existing evidence and guides the targeted generation of new data, where required, to inform regulatory decision-making regarding potential hazard and/or risk. Within an IATA, data from various information sources (i.e. physicochemical properties, in silico models, grouping and read-across approaches, in vitro methods, in vivo tests and human data) are evaluated and integrated to draw conclusions on the hazard and/or risk of chemicals.” In other words, the information sources within IATA typically include a variety of model predictions.

One of the first IATA formally endorsed at the OECD deals with skin corrosion and irritation [8] while more recent IATA projects cover a variety of endpoints including skin sensitisation, non-genotoxic carcinogenicity and developmental neurotoxicity. In addition to the development of formal guidance, the IATA Case Studies Project (http://www.oecd.org/chemicalsafety/risk-assessment/iata-integrated-approaches-to-testing-and-assessment.htm) is an ongoing OECD effort to increase experience with the use of IATA by developing case studies on specific chemicals or groups of chemicals. The aim is to create a common understanding of the use of novel methodologies as well as the considerations and guidance stemming from the case studies.

### 2.2 Quantitative structure–activity relationships (QSARs)

Principles for establishing the validity of QSARs for regulatory purposes were first proposed during an ICCA-LRI workshop held in Setubal, Portugal, in 2002 [15]. The so-called “Setubal principles” were subsequently adopted by the OECD, with some modifications, and became the “OECD principles for (Q) SAR validation for regulatory purposes” [16]. According to these principles, these models should be associated with the following information: 1) a defined endpoint; 2) an unambiguous algorithm; 3) a defined domain of applicability; 4) appropriate measures of goodness-of-fit, robustness and predictivity; and 5) a mechanistic interpretation.

Practical guidance on how to apply the QSAR validation principles was subsequently developed by the OECD [17]. This has also informed the ECHA guidance for REACH [18] and the EFSA guidance on the dietary risk assessment of pesticide residues [19].

### 2.3 Defined approaches for skin sensitisation

As mentioned above, IATA consist of multiple methods used to carry out a safety assessment and reach a conclusion. The information used within an IATA is heterogeneous, ranging from existing data and expert judgements (e.g. read-across arguments) to predictions from in vitro and/or in silico models. In cases where data is derived from multiple models and then combined using a rule-based or mathematical Data Interpretation Procedure (DIP), the term Defined Approach (DA) has been coined. A DA consists of a fixed set of data/information sources (typically methods or models) along with an algorithm that interprets the data, generally in the form of a toxicity prediction.

Guidance on defined approaches focused initially on the prediction of skin sensitisation [8], [14], [20]. The principles laid down in these documents were subsequently discussed by the International Cooperation on Alternative Test Methods [21], leading to an ongoing project within the OECD to develop a guideline for skin sensitisation DAs.

### 2.4 In vitro developmental neurotoxicity methods

A framework for assessing the “readiness” of in vitro methods for developmental neurotoxicity (DNT) has been developed and is being used to support an EU (EFSA)-led OECD project aimed at developing IATA for DNT [22]. DNT is an endpoint of high health concern, and one for which the underlying mechanisms of toxicity are reasonably well established. At the same time, in vivo benchmark data are scarce, and have been generated by rodent methods that do not capture all of the mechanisms in humans, and are relatively insensitive (miss toxic effects) compared with in vitro models.

The resulting framework is probably the most elaborate one developed by the in vitro community. It consists of 15 criteria, each of which is subdivided into several subcriteria. In addition, a scoring system was devised by weighting the criteria. This was applied to 17 in vitro methods to rank them in terms of their readiness. The final ranking is categorical, with four levels of readiness. The readiness ranking is used to decide on the possible applications of the methods, while the detailed scoring is used to inform method refinement.

### 2.5 Physiologically based kinetic (PBK) models

Building on previous guidance developed by the World Health Organization [7], a recent project at the OECD has developed a PBK Model Assessment Framework. This has been designed to capture not only traditional “familiar” uncertainties, but also the additional “unfamiliar” uncertainties resulting from the use of in vitro and in silico methods in calibrating model parameters. The guidance also addresses situations where reliable in vivo kinetic data are lacking for model development and predictivity assessment.

The framework includes two categories of considerations in addressing the “adequacy” of a model for a given application. In this context, adequacy refers to the usefulness of the information for a specific regulatory purpose (e.g. risk assessment), in line with ECHA guidance [23].

The first category is “Context & Implementation”, which considers the regulatory application and context of use, which together determine the degree of acceptable uncertainty. Additional contextual factors include evidence of code availability and peer input/review, since these factors additionally influence the degree of confidence in a model.

The second category is “Model Validity”, which addresses confidence in the model based on its intrinsic validation characteristics. Model validity consists of five main considerations, the first four of which do not depend on the use of in vivo data. These considerations (see also Table 1) are: 1) biological basis; 2) theoretical basis of model equations; 3) reliability of input parameters; 4) sensitivity of output to input parameters; and 5) goodness-of-fit and predictivity.

### 2.6 Computational models outside of toxicology

Although not directly related to toxicology, there is also extensive relevant literature on the verification and validation of computer simulations [24], [25], including verification and validation methods [26], [27] and on how to build credible simulation models [28]. Another important recent development has been the verification and validation of computational models in the field of in silico medicine, such as in silico trials of new and repurposed drugs [29], [30].

### 2.7 Similarities and differences between the frameworks

Comparing the different frameworks in Table 1, it can be seen that they share a lot of common features. They all break down the two major uncertainties relating to predictive toxicology approaches, namely reliability and relevance, in different ways. They all express the need to clearly define the test system or model, and to characterise its reliability (reproducibility). Equally, they all embrace the need to demonstrate relevance, which is partly related to biological relevance of the test system or model, and partly to the ability to predict the toxicological endpoint of interest.

At the same time, certain attributes are explicit in some frameworks yet implicit in others. For example, the QSAR community emphasises the need for a “defined endpoint”, since QSAR modellers had a tendency to aggregate data generated by different protocols (to make best use of scarce data) whereas regulatory assessors would expect data from a standardised protocol and generally a specific test guideline. Similarly, the QSAR community was conscious that QSAR models are typically applicable to a relatively narrow range of chemical classes, so they introduced the concept of the applicability domain. On the other hand, the QSAR community did not explicitly consider the need for reliability assessment, in the sense of reproducibility, since this was considered a given for software tools. The need for a defined endpoint was implicitly covered by test definition for the in vitro methods community, while the concept of application domain was taken over from the QSAR domain later on.

The frameworks also differ in terms of the emphasis they place on predictive capacity. Defined approaches for skin sensitisation have evolved in a context that is not only data rich (i.e. availability of skin sensitisation data in animals and humans), but also well understood in mechanistic terms. The DNT situation is different, since in vivo data for this endpoint are scare and, in any case, considered to lack human relevance. Thus, the credibility of these in vitro tests is based mainly on their mechanistic relevance to human biology, as well as thorough characterisation of the test method in line with good in vitro method practices [31]. In this case, the predictive capacity cannot be really demonstrated as such, but nor is this considered necessary.

## 3 Factors underpinning scientific credibility

By its nature, validation does not guarantee acceptance of new approaches by decision-makers since acceptance depends on many non-scientific and situation-specific considerations including economic, political and psychological factors [32], [33].

To better relate validation with acceptance, it is helpful to consider the bridging concept of scientific credibility, defined by Schruben as “the willingness of persons to base decisions on information obtained from the model” [34]. One can consider validation as an important, or even essential, step in establishing scientific credibility [35], [36], but not necessarily sufficient in itself. Put another way, a lack of credibility can lead to the rejection of valid predictions [36], [37].

In the pursuit of establishing credibility and gaining acceptance of new approaches in toxicology, it is useful therefore to review validation principles that have emerged in different communities over the years and to consider them in context of a set of method-agnostic factors underpinning scientific credibility. These factors (Table 2) have already been proposed and applied in a very different context, namely the validation of complex multi-physics engineering simulation models [38]. In the latter publication, two additional credibility factors (“theoretical ancestry” and “model building credentials”) were included to capture properties of “self-vindicating models”, i.e. models for which there is no quantitative or qualitative observational evidence. In these cases, credibility would be based on the theoretical ancestry of the underlying physical models, as well as accepted modelling praxis. These factors were omitted from Table 2, Table 3 since they are rarely considered in predictive toxicology, at least in its current stage of theoretical development.

**Table 2:** Credibility factors and potential strategies to demonstrate that a predictive method possesses them.

No^1^	Credibility Factor	Explanation	Potential strategies to demonstrate them
1	Assumption Confirmation	Assumptions and their limitations identified and justified by empirical evidence	a) Identify assumptions underpinning approach & their limitations
			b) Collate observational evidence to justify each assumption
			c) Identify [characterise] uncertainty associated with assumptions
2	Qualitative concordance	Extent to which predicted behaviour matches observations	a) Assess extent to which predicted behavioural trends [effects] match observations of behaviour
			b) Assess uncertainty for behavioural trends
3	Quantitative concordance	Extent to which predictions represent observational data	a) Quantify the extent to which predictions represent observational data for the intended use of the approach
			b) Quantify uncertainty in the quantitative predictions
4	Explanatory power	Ability to explain phenomena, behaviour and situations including those on which the predictive approach is based, as well as those on which it is not based	a) Demonstrate predictions explain observed phenomena and behaviour [effects]
			b) Use predictive approach to explain situations & effects other than those on which the approach is based
			c) Identify limitations in explanatory power & discrepancies with biological plausibility
5	Internal consistency	Lack of logical contradictions	a) Demonstrate approach predicts already known result [e.g. training dataset] (calibration)
			b) Demonstrate perturbation of input parameters produces expected result (sensitivity)
			c) Demonstrate predicted behaviour [effects] disappears in appropriate circumstances (disappearance)
			d) Demonstrate predictions unchanged by elimination of all plausible sources error (artefact removal)
			e) Assess reproducibility of approach in different environments
6	External consistency	Ability to predict similar effects/behaviour as alternative approaches	a) Predict similar effect/behaviour with other approach[es] (external comparison)
			b) Quantify uncertainty in reproducibility and other prediction comparisons
7	Simplicity	Minimum level of complexity required to generate predictions that accurately represent the real-world	a) Build a strong narrative with appropriate level of detail that is both precise and concise
			b) Demonstrate appropriate degree of simplicity by removal of each core assumption [element] producing a significant change in prediction
			c) Express uncertainty about level of simplicity and influence of known unknowns and unknown unknowns

1 Factors 1–3 are largely data-based, from Naylor and Finger [39], while factors 4–7 are largely knowledge-based, from McMullin [40].

Table 2, Table 3 highlight the utility of the credibility factors as a framework to identify commonalities and differences between current approaches. The framework could be used to explore strengths and possible weaknesses, and ensure that future validation strategies are fit for purpose and applicable to the new generation of predictive toxicology approaches. The framework is intended to be flexible – according to the context (availability of data and knowledge, impact of decision-making), a greater or lesser emphasis might be placed on a given factor.

Two main types of credibility factor can be distinguished: those that rely primarily on data (data-based factors) and those based primarily on knowledge (knowledge-based or epistemic factors). In Table 2, factors 1–3 are more data-based, and derived from Naylor and Finger [39], while factors 4–7 are more knowledge-based, derived from McMullin [40].

In the ideal situation, validation is based on both types of factors, but one can compensate for the other to some degree. Particular challenges in toxicology are that there are situations where there is reasonable mechanistic knowledge but relatively little in vivo data (e.g. DNT), or there is (potentially) a sufficient amount of in vivo data, but relatively little understanding of underlying biological mechanisms (e.g. long term, transgenerational effects of chemicals).

### 3.1 Data-based factors

When sufficient, high-quality data are available, the comparison of the predictive approach to the system can be performed using data in the form of detailed measurements of the system whose behaviour the approach is intended to represent. This should consist of: (1) confirming the appropriateness of the underpinning assumptions, which can be based on existing knowledge of the components and processes within the system; (2) assessing the qualitative concordance of the prediction; and if the data strength permits, (3) evaluating the quantitative concordance of the prediction. These three steps provide the first three credibility factors listed in Table 2 together with potential strategies to demonstrate that a predictive approach possesses them and hence yields credible predictions.

In more detail, for (1) it is appropriate to first identify and catalogue the assumptions involved, to collate the observational evidence that justifies the use of each assumption and to identify and characterise the uncertainty associated with each assumption. For each of the concordance factors in (2) and (3), there are two strategies proposed, namely: assessing the extent to which the predictions and observations match, either qualitative or quantitative; and quantifying the uncertainty associated with the predictions. Whenever possible, the techniques of quantitative concordance [e.g. 41] should be used but when this is not viable then some form of qualitative concordance should be undertaken. In the extreme, it is even possible to provide a relative error for the prediction expressed as a probability that the prediction belongs to the same population as measured data [42]. However, it is more likely that the intrinsic uncontrollability of real-world biological phenomena and the difficulty of capturing the complexity of the biological system will limit the quantity and quality of data available for comparison with predictions.

### 3.2 Knowledge-based factors

When there is sufficient knowledge of the underlying biology, knowledge-based factors can be applied. TS Kuhn [43] identified five attributes of a good theory: accuracy within its domain; consistency; broad scope; simple and fruitful. McMullin [40] proposed that there is a set of tacit values in modern science that augment ‘the truth-like nature of science’ and reworked Kuhn’s attributes by separating consistency into internal and external consistency and by redefining ‘broad scope and fruitful’ as ‘unifying power and fertility’. In the context of climate change modelling, where predictions are required in the far-term time domain with only sparse data available in the near-term time domain, Biddle and Winsberg [44] have proposed that predictions are more likely to be true when they are based on models with strong epistemic properties. Epistemic properties include: explanatory power; internal consistency; external consistency; and simplicity [45]. Hence, a knowledge-based assessment of a predictive toxicology approach should include: (4) demonstrations of its explanatory power; (5) evidence of the reproducibility and parametric sensitivity of the predictive approach as an indication of its internal consistency; (6) comparisons with other sources of knowledge to demonstrate external consistency; and (7) information about the simplicity of the predictive approach relative to other approaches.

Explanatory power is an obvious property of a reliable and credible predictive approach because those that lack explanatory power, i.e. the ability to elucidate and describe observed or known phenomena, are likely to be discarded anyway. Bailey [46] has highlighted that the plausibility of models is enhanced when they can describe, in a consistent fashion, situations different from the original ones they were intended to represent. In other words, the more extensive the explanatory power of a predictive approach, the more likely decision-makers are to trust its results.

A set of strategies for demonstrating that a predictive approach possesses the knowledge-based factors is provided in Table 2. For explanatory power, these strategies include: (a) demonstrating that the outputs from a predictive approach explain observed phenomena and behavioural effects; (b) using the predictive approach to explain situations and effects other than those used in designing and developing the approach; and (c) identifying limitations in the explanatory power of the predictive approach and any deviations from biological plausibility. Since these strategies might be deployed in the absence of empirical data describing the real-world effect of interest, each of these strategies could be deployed using qualitative observations or by identification and recording of trends.

The final epistemic property in Table 2 is simplicity. Decision-makers are also more likely to trust the outputs of a predictive toxicology approach for which they have some level of understanding. Law [28] lists understanding of, and agreement with, modelling assumptions as a factor that will increase credibility. Hence, since simpler models are easier to understand, simplicity is directly related to credibility and might therefore involve building a strong narrative with the appropriate level of detail, i.e. it is both concise and relevant [47], so that it creates an engaging and coherent story. Stories that organise information without internal contradictions and that are compatible with common assumptions are more compelling [48]; and coherent stories are easier to process than incoherent ones [49] and thus more likely to be seen as credible. Often, a predictive approach will bond together disparate storylines that develop in the mental models and hypotheses of decision-makers as they are confronted with evidence and information from multiple sources; and hence the narrative needs to connect to the wider context in which the decision-maker is functioning [50]. Hence, building a strong narrative with the appropriate level of detail that is both precise and concise is one strategy for demonstrating that the predictive approach has an appropriate level of simplicity, thereby increasing the prospect of it being considered as credible. In this context, toxicological models are more likely to be credible if they capture established knowledge of the underlying mechanisms of chemical toxicity, as expressed for example in Adverse Outcome Pathways (http://www.oecd.org/chemicalsafety/testing/projects-adverse-outcome-pathways.htm).

A more philosophical argument can be made in favour of simplicity; namely, replacing a simplifying, but inaccurate, assumption or hypothesis by a more complex one should not result in a more reliable or realistic prediction [51], [52]. Such simplifying assumptions have been labelled by Kuorikoski et al [53] as tractability assumptions because they make predictions tractable or viable, in contrast to substantial assumptions that define the causal core of the predictive approach. Hence, a further step in demonstrating that a predictive approach has an appropriate level of simplicity is to show that removal of any core assumption produces a significant change in the predictions. It has been recognised that the interdependence of assumptions might make removal and substitution of individual assumptions impractical [54]. However, when such a process is viable, it might assist in the third strategy associated with simplicity, which is identifying and characterising the uncertainty associated with the level of simplicity and the influence of unknowns.

Considerations of the credibility factors based on the strategies described in Table 2 can be collated into evidence profiles, which Guyatt et al [55] believe are helpful to decision-makers for delivering succinct, transparent and easily-digestible summaries of evidence. Sargent [24], in the context of verification and validation of simulations, states that both summary and detailed documentation is required. Similarly, the exposure modelling community has concluded there is a need for different levels of model description [56]. The recently developed OECD template for reporting PBK models also includes a high-level summary of the model and its intended application.

## 4 Analysis of selected validation frameworks in terms of credibility factors

To illustrate how the credibility factors can be applied in toxicology, we have used them to characterise four of the more recent validation frameworks, namely QSAR models, defined approaches, in vitro DNT methods, and physiologically based kinetic models (Table 3).

**Table 3:** Relationship between different validation frameworks in terms of the credibility factors.

No	Credibility Factor	QSAR models	Defined approaches	In vitro DNT methods	PBK models
1	Assumption Confirmation	Mechanistic interpretation	Relevance	Test system: human relevance of biological system Applicability domain: link to adverse outcome pathway	Biological basis Theoretical basis of model equations
2	Qualitative concordance	Goodness-of-fit, predictive capacity	Predictive capacity	Prediction model	Goodness-of-fit, predictive capacity
3	Quantitative concordance	Goodness-of-fit, predictive capacity	Predictive capacity	Prediction model	Goodness-of-fit, predictive capacity
4	Explanatory power	Goodness-of-fit, predictive capacity, applicability domain	Predictive capacity Applicability domain	Prediction model Applicability domain	Goodness-of-fit, predictive capacity
5	Internal consistency	Goodness-of-fit, robustness	Goodness-of-fit	Test method controls Data evaluation Robustness Test benchmarks	Sensitivity of output to parameters
6	External consistency	Concordance of prediction with other QSARs	Concordance of prediction with other defined approaches	Testing strategy. Concordance of prediction with other in vitro DNT methods	Concordance of prediction with other PBK models
7	Simplicity	Number and nature of descriptors	Complexity	Not addressed	Model structure. Conceptual model
	**Other considerations**	Defined endpoint	Transparency – availability of elements	Documentation Screening hits	Regulatory application and context of use Code availability Peer input/review

It can be seen that the mapping between credibility factors and validation principles is not necessarily one-to-one. There are also validation principles or assessment elements that are not directly associated with the current list of credibility factors. For example, the assessment framework for defined approaches considers the practical availability of the elements (component parts) of the predictive approach. This would also include then an assessment of proprietary elements and fairness of access in a commercial sense. Similarly, the PBK framework includes considerations such as availability of model code (to enable independent implementation and verification of the model), and the extent of peer engagement in the model development process.

## 5 Concluding remarks

In recent years, there has been increasing recognition that while the principles of validation are sound, the practical validation of new methods needs to change, in order to adapt to the increasing number and variety of predictive approaches [57].

Burgdorf et al [58] recognised that relevance is much more difficult to demonstrate than reliability, and they argue this should be done from the perspective of IATA, rather than stand-alone test methods. They emphasise that validation should be knowledge-based (even though this was always the case), flexible (again this was always the case), and iterative (admittedly, this has proven difficult, given the institutional mechanisms of formal validation and acceptance). They also stress the importance of multi-stakeholder involvement (see also below) and the utility of case studies in building confidence in new approaches and technologies.

With a view to developing a systematic approach to evaluate predictive approaches and determining their suitability for regulatory application, Parish et al [59] have proposed a three-step evaluation framework. The first step is to determine how the method will be used in relation to three main contexts-of-use (prioritisation, hazard screening, or risk assessment). Step 2 is a set of core principles, irrespective of the context-of-use, that must to be addressed, while Step 3 lays out criteria that will vary in their level of importance based on the context-of-use. Associated with these steps, 15 evaluation criteria are proposed. Fourteen of these criteria refer to “intrinsic” characteristics of the test system, while one of them is “extrinsic” referring to the existence of an independent peer review process. Overall, these criteria are more applicable to experimental methods, rather than computational ones. It should also be noted that the three contexts-of-use are more meaningful in a US regulatory setting than in the EU regulatory environment. The core of the EU regulatory framework is centred on hazard classification (based on formal criteria) which in some sectors is supplemented with a risk assessment. Prioritisation does not play a role, or at least not a legally binding one. In addition, risk–benefit considerations also apply in a few sectors, e.g. pharmaceuticals.

As a result of the evolution of validation practices over the past 25 years, the regulatory community is now confronted not only with an increasingly diverse and sophisticated range of new methods, but also a bewildering array of assessment frameworks. Moreover, a crucial gap in the regulatory guidance literature is an assessment framework that not only identifies and characterises the uncertainties associated with the overall assessment (regulatory conclusion), but also traces how these uncertainties propagate from those of the underlying methods and test data. The development of such an over-arching framework will be very challenging, and will need to reconcile the multiplicity of method-centric frameworks. It is argued here that the credibility factors could serve as a comparative framework, or system of equivalences [12], to aid cross-disciplinary communication and harmonisation. Further work could test the utility of these credibility factors to a broader range of case studies, such as those developed within the OECD IATA Case Studies Project.

It is important to note that model credibility is not the exclusive belief of the developer but should be a shared trust with the decision-maker. The shared nature of scientific credibility implies that it needs to be established through a process of social epistemology [60], [61] which ideally involves personal interactions between all key actors. It is proposed here that practising social epistemology throughout the lifecycle of a predictive approach will lead to a shared knowledge of the salient features of the approach, its strengths and limitations, and a common understanding of how it might be best applied. In regulatory toxicology, this is still the exception rather the rule, although there are signs that this is changing. The ongoing OECD work on DNT and IATA case studies provide good examples.

In conclusion, the process of social epistemology can promote confidence, transparency, trust and ultimately acceptance of new predictive toxicology approaches. However, this also requires the use of a common language, or at least a means of comparing and contrasting the validation practises of different scientific communities. In this commentary, we have proposed the use of credibility factors as the basis of such a comparative framework.

## Declaration of Competing Interest

The authors declare that they have no known competing financial interests or personal relationships that could have appeared to influence the work reported in this paper.
